# Lipoprotein(a) in Cardiovascular Diseases and Emerging Therapeutic Strategies

**DOI:** 10.1007/s10557-025-07810-1

**Published:** 2025-11-20

**Authors:** Rami A. Al-Horani, Alexandra C. Selico-Dunn, Emily Lauren Schenk Smith

**Affiliations:** 1 Division of Basic Pharmaceutical Sciences, College of Pharmacy, Xavier University of Louisiana, New Orleans, LA 70125 USA; 2 College of Pharmacy, 1 Drexel Drive, New Orleans, LA 70125-1089 USA

**Keywords:** Lipoprotein(a), ASCVD, Pelacarsen, Olpasiran, Zerlasiran, Lepodisiran, Muvalaplin, Obicetrapib, SiRNA, Antisense oligonucleotides

## Abstract

**Purpose:**

Lipoprotein(a) [Lp(a)] is increasingly recognized as a genetically determined, independent risk factor for atherosclerotic cardiovascular disease (ASCVD). This review examines the structure, pathophysiology, and epidemiology of Lp(a), with a focus on its contribution to ASCVD and related conditions such as aortic valve stenosis and peripheral artery disease. The main research question addresses how Lp(a) influences cardiovascular risk and how emerging therapies may modify this risk.

**Methods:**

This review synthesizes published evidence describing the biological characteristics of Lp(a), its mechanistic roles in disease, and its epidemiologic associations with cardiovascular outcomes. It also evaluates current and investigational therapeutic approaches by examining clinical trial data for agents targeting Lp(a).

**Results:**

Lp(a) contributes to residual cardiovascular risk through proatherogenic, proinflammatory, and prothrombotic mechanisms. Current evidence highlights its involvement in ASCVD, aortic valve stenosis, and peripheral artery disease. Clinical studies of antisense oligonucleotides, small interfering RNAs, oral small molecules, and CRISPR-based gene editing, including pelacarsen, olpasiran, zerlasiran, lepodisiran, muvalaplin, and obicetrapib, demonstrate promising efficacy and safety. These agents show potential to significantly reduce Lp(a) levels and influence future cardiovascular prevention strategies.

**Conclusion:**

As novel therapies advance and clinical guidelines evolve, Lp(a) is emerging as a central determinant in personalized cardiovascular care. The increasing emphasis on Lp(a) testing underscores its importance in risk stratification and future therapeutic decisionmaking.

## Introduction

Atherosclerotic cardiovascular disease (ASCVD) remains one of the leading causes of morbidity and mortality worldwide, placing a heavy economic burden on healthcare systems [1]. Despite significant progress in both primary and secondary prevention, many patients continue to experience adverse cardiovascular events due to what is termed “residual cardiovascular risk” [2]. Research has identified thrombosis, dysregulated cholesterol metabolism, and chronic inflammation as key drivers of this persistent risk [2, 3]. Within this framework, lipoprotein(a) [Lp(a)] has been recognized as an independent and causal risk factor for ASCVD [4, 5].

Lp(a) promotes atherosclerosis and its complications through multiple mechanisms, including the carriage of proatherogenic molecules such as oxidized phospholipids, as well as the induction of inflammation and thrombosis [6]. Elevated Lp(a) levels are causally linked to worse cardiovascular outcomes, and emerging evidence suggests that lowering Lp(a) can help reduce complications in ASCVD and related conditions, including aortic valve calcification and stenosis [7–9].

Awareness of Lp(a)’s clinical importance, however, remains limited, and routine testing has not been widely adopted [10]. This is partly due to the absence of approved pharmacological therapies that specifically target Lp(a). Nevertheless, several investigational agents with potent Lp(a)-lowering capacity and favorable safety profiles are currently in clinical development, representing a potential breakthrough for patients with elevated levels [11]. The extent to which these therapies will reduce ASCVD risk and improve long-term outcomes remains under active investigation.

Clinical guidelines classify Lp(a) as a genetically determined, atherogenic variant of LDL and emphasize its role as a biomarker of inherited cardiovascular risk. Because Lp(a) concentrations are largely stable throughout life in the absence of targeted interventions, levels ≥ 50 mg/dL (125 nmol/L) are typically considered elevated. Even without approved therapies, a single lifetime Lp(a) measurement is often considered cost-effective for risk assessment in patients with ASCVD risk factors [12]. However, recent evidence suggests that this approach may not always be sufficient. A study of nearly 12,000 adults across three Mayo Clinic sites showed that while most patients with clearly normal or high Lp(a) levels remained stable, nearly half of those with borderline concentrations (30–50 mg/dL) shifted categories on repeat testing. Moreover, about one-quarter of these individuals demonstrated changes greater than 10 mg/dL, with variability more common among women, patients with ASCVD, those with LDL-C ≥ 100 mg/dL, and those on statin therapy. These findings indicate that patients with borderline Lp(a) levels may benefit from repeat testing to ensure accurate risk stratification [13].

Evidence from a large UK database analysis has further strengthened the association between elevated Lp(a) levels (> 70 mg/dL or 150 nmol/L) and extra-coronary ASCVD, including peripheral artery disease and carotid artery stenosis. Among patients with peripheral artery disease, elevated Lp(a) was linked to a 57% higher risk of major adverse limb events, while those with carotid stenosis faced a 40% increased risk of stroke. Modeling studies suggest that lowering Lp(a) by 35 mg/dL (75 nmol/L) could reduce the risk of incident peripheral artery disease by 18% and carotid stenosis by 17% [14].

Despite these strong associations, therapeutic options for lowering Lp(a) remain limited. Niacin has been evaluated as a potential intervention but is rarely used in practice. In the UK analysis, only 77 of more than 460,000 participants were taking niacin. Furthermore, large-scale trials, including AIM-HIGH [15] and HPS2-THRIVE [16], failed to demonstrate cardiovascular benefit, even though AIM-HIGH participants experienced a 25% reduction in Lp(a). These findings have contributed to the decline of niacin as a therapeutic option for Lp(a) management.

Another critical consideration is the independence of Lp(a) from LDL-C. Statin therapy, while highly effective in lowering LDL-C, does not mitigate the cardiovascular risk conferred by elevated Lp(a). A 2024 meta-analysis confirmed that Lp(a) acts as an independent risk factor irrespective of achieved LDL-C levels. Notably, patients with Lp(a) > 50 mg/dL and LDL-C in the lowest quartile (3.1–77 mg/dL) still carried a 38% higher ASCVD risk compared with those with lower Lp(a) concentrations [6]. This aligns with earlier research demonstrating that statin-mediated LDL-C reduction does not diminish Lp(a)-associated risk [17].

This review synthesizes the current evidence linking Lp(a) with ASCVD, explores emerging Lp(a)-lowering therapies, and considers their potential role in both primary and secondary prevention. It also discusses existing strategies for cardiovascular risk reduction and management for patients with elevated Lp(a), pending the availability of targeted therapies currently under clinical investigation. This review was conducted in a narrative style. Literature was identified through PubMed, Google Scholar, and ClinicalTrials.gov up to the date of the first submission, using search terms including lipoprotein(a), Lp(a), cardiovascular risk, antisense oligonucleotide, short-interfering RNA (siRNA), CRISPR, and cholesteryl ester transfer protein (CETP) inhibitors. We prioritized clinical trials, large observational studies, guideline statements, and recent systematic reviews. Additional references were identified by reviewing the bibliographies of key articles. Because this was a narrative rather than a systematic review, no formal inclusion/exclusion criteria were applied.

## Lp(a): Structure, Level, and Pathophysiology

Lipoproteins are complexes of lipids and proteins that transport cholesterol and triglycerides in the blood. Very-low-density lipoproteins (VLDL) are produced by the liver and mainly carry triglycerides, which are progressively metabolized into intermediate-density lipoproteins (IDL) and then low-density lipoproteins (LDL), the major carriers of cholesterol and a key driver of ASCVD. Lp(a) resembles LDL but has an additional apolipoprotein(a) attached, giving it both atherogenic and prothrombotic properties, thus adding independent ASCVD risk beyond LDL cholesterol. In contrast, high-density lipoproteins (HDL) are involved in reverse cholesterol transport, shuttling cholesterol from peripheral tissues back to the liver, and are generally considered protective. Understanding Lp(a) within this lipoprotein family highlights why it is increasingly recognized as a distinct, causal risk factor for ASCVD [18].

**Fig. 1:**
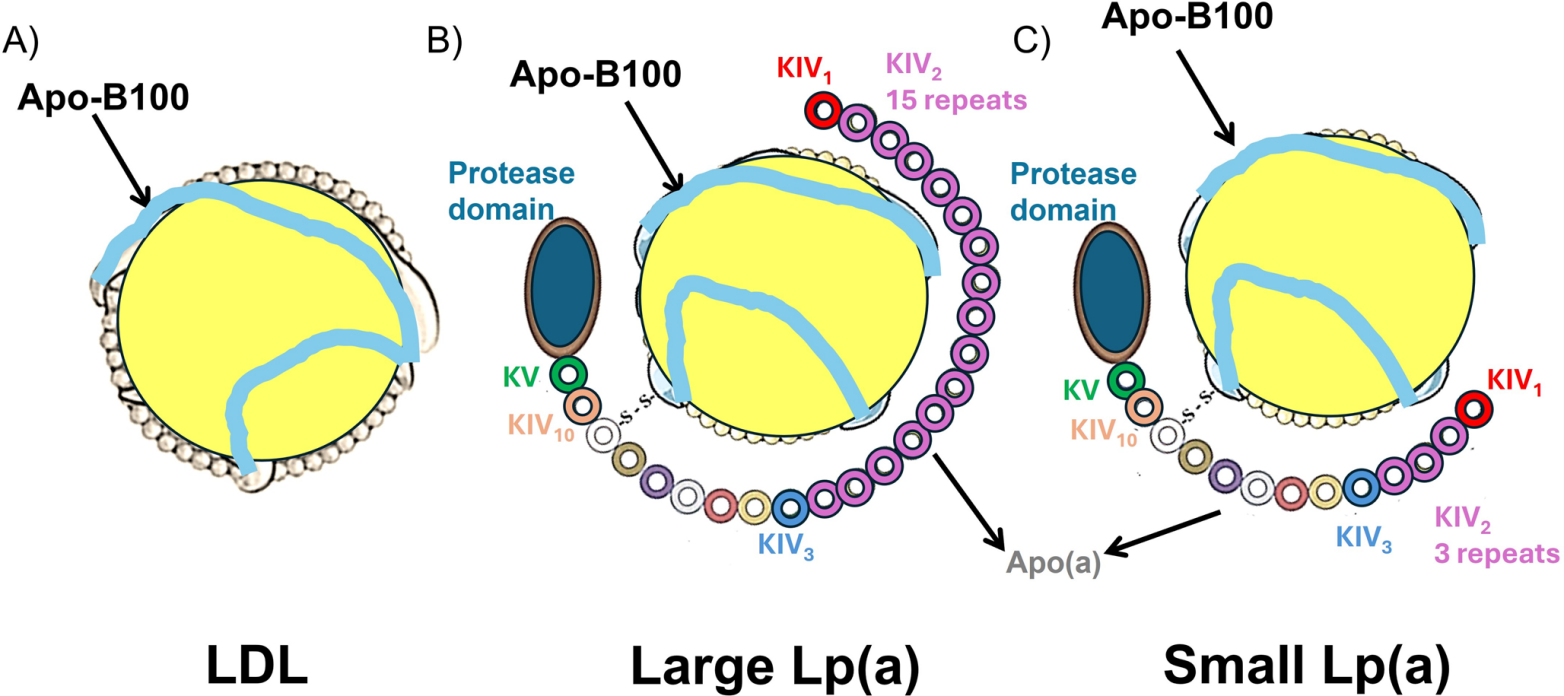
The structures of LDL (**A**), small and large Lp(a) (**B** and **C**). Human Lp(a) consists of an LDL-like particle and an apoB-100, to which glycoprotein apo(a) is disulfide-linked. Apo(a) contains 10 different types of kringle IV domains (KIVs), composed of 1 copy of KIV1, multiple copies of KIV2, and 1 copy of KIV3-10, and a single kringle V domain followed by an inactive protease domain. KIV2 repeats of apo(a) determine the size of different Lp(a) isoforms

Lp(a) is a genetically determined variant of LDL cholesterol, encoded by the *LPA* gene and synthesized in the liver. Structurally, it consists of apolipoprotein B100 (apoB100) covalently linked to apolipoprotein(a) [apo(a)] [19]. Apo(a), which is also encoded by *LPA*, wraps around the LDL-like particle, forming a hydrophilic outer layer composed of multiple three-dimensional, heavily glycosylated loops of 80–90 amino acids known as kringles. These structural motifs are grouped into two main domains: kringle IV (KIV) and kringle V (Fig. 1). The KIV domain contains 10 distinct subtypes (KIV1–KIV10), with KIV2 typically present in multiple tandem repeats, ranging from 1 to > 40. Variation in subtype composition and KIV2 repeat number gives rise to diverse Lp(a) isoforms. Smaller isoforms (10–22 KIV repeats) and larger isoforms (> 22 repeats) differ not only in molecular size but also in plasma concentration [1]. Smaller isoforms are clinically significant because they are produced in greater molar quantities over time, thereby conferring a higher cardiovascular risk [20].

Circulating Lp(a) concentrations vary widely between individuals, from 0.2 to 750 nmol/L (0.1–300 mg/dL) [21]. A genome-wide association meta-analysis identified 31 single-nucleotide polymorphisms (SNPs) independently associated with Lp(a) levels after adjustment for apo(a) isoform size. Cardiovascular risk increases in a roughly linear fashion with rising Lp(a), similar to LDL cholesterol. However, consensus thresholds remain debated: levels above 30 mg/dL have been associated with increased risk, while values of 100–125 nmol/L (≈ 50 mg/dL) are often proposed to define “elevated” Lp(a) [22, 23].

Globally, an estimated 20–30% of the population, about 2 billion people, have elevated Lp(a), though prevalence varies depending on ancestry, sex, geographic region, and the threshold applied. Concentrations are primarily genetically determined, with genotype accounting for ~ 90% of interindividual variability. Adult levels are usually established by age five, though they may continue to rise later in life. Non-genetic influences such as chronic illness, hormonal changes, or lifestyle factors can also affect concentrations [24]. Women generally have 5–10% higher Lp(a) levels than men, with an additional rise observed after menopause [25].

Ancestry strongly affects Lp(a) distribution, allele frequencies, and the relationship between apo(a) size and plasma levels [26]. Black and South Asian individuals tend to have the highest concentrations, whereas East Asian and Hispanic individuals often have lower levels than White individuals [27, 28]. Median Lp(a) concentrations are about three times higher in Black individuals compared with White individuals, although the link between elevated Lp(a) and cardiovascular risk appears consistent across populations [26, 29]. Consequently, ancestry-specific cutoff values for elevated Lp(a) have not been recommended.

**Fig. 2:**
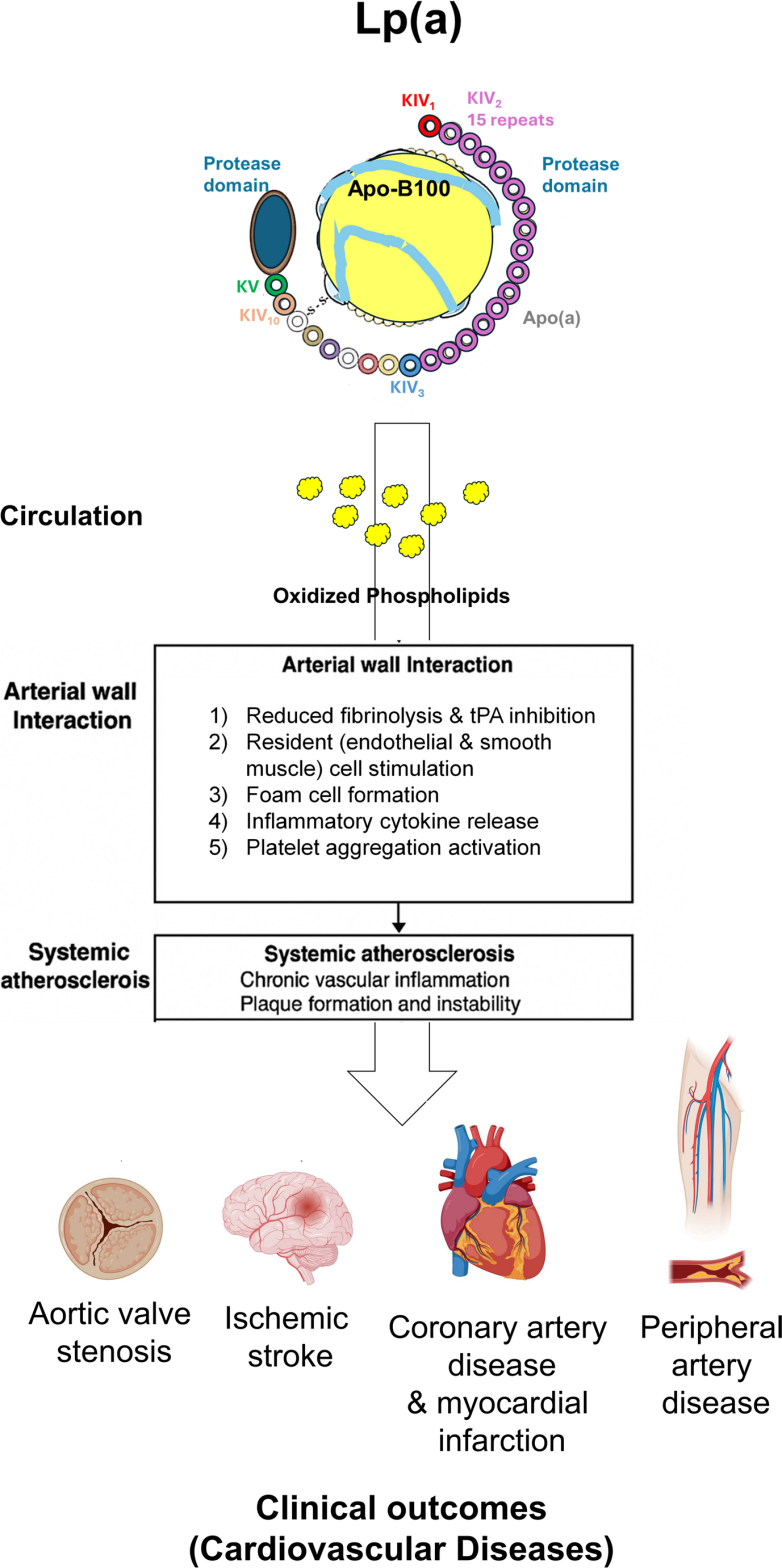
The role of lipoprotein(**a**) in systemic atherosclerosis and cardiovascular diseases

Importantly, although the normal physiological role of Lp(a) remains unclear, extensive mechanistic, epidemiological, and genetic evidence supports its causal involvement in several cardiovascular conditions, including coronary and peripheral artery disease, heart failure, aortic valve stenosis, aortic disease, and ischemic stroke (Fig. 2) [7, 8, 30–36]. Large-scale analyses, such as the Emerging Risk Factors Collaboration (126,634 participants from 36 biomarker studies), have confirmed that elevated Lp(a) is associated with a higher risk of fatal and first-time nonfatal vascular events, particularly coronary artery disease and ischemic stroke [30]. Mendelian randomization studies further validate the causal role of Lp(a) across populations, including individuals with familial hypercholesterolemia and established coronary disease [7, 8, 22, 31].

Genetic studies have also linked Lp(a) to aortic valve calcification and stenosis, demonstrating associations with single-nucleotide polymorphisms in the *LPA* gene and kringle IV repeat polymorphisms [34, 37]. Elevated Lp(a) levels, along with oxidized phospholipids, are consistently associated with greater incidence, prevalence, and progression of aortic calcification and stenosis [38]. Furthermore, cardiac MRI data indicate that high Lp(a) is related to subclinical myocardial fibrosis, scar formation, and left atrial remodeling [39].

Lp(a) contributes to cardiovascular risk through proatherogenic, proinflammatory, and prothrombotic mechanisms [21, 40]. On a molar basis, it is more atherogenic than LDL cholesterol because it combines LDL components with apo(a) [41]. Its markedly greater atherogenicity is largely attributed to its high content of oxidized phospholipids [42, 43]. Lp(a) preferentially deposits at sites of vascular injury rich in lysine-binding domains, where it promotes endothelial adhesion molecule expression, macrophage recruitment, and foam cell formation [40, 44, 45].

Although Lp(a) levels are genetically determined, they may increase in inflammatory states such as rheumatoid arthritis and chronic kidney disease [46]. The connection between Lp(a) and inflammation is further supported by clinical evidence that IL-6 inhibition with ziltivekimab reduces Lp(a) levels by 16–25% in a dose-dependent fashion [47]. Elevated Lp(a) also promotes the formation of rupture-prone coronary plaques characterized by necrotic cores and thin fibrous caps, particularly in proinflammatory environments [44]. Research also confirms that Lp(a) levels can fluctuate and increase following a myocardial infarction. This transient elevation is believed to be a response to the inflammatory state caused by the heart attack [48].

Beyond atherogenesis, Lp(a) augments atherothrombosis through apo(a)-mediated activation of platelet protease-activated receptor-1 (PAR-1) and interference with fibrinolysis, owing to its structural homology with plasminogen. It may also accelerate the progression of coronary and peripheral atherosclerosis by fostering fibrin deposition and accumulating within vulnerable plaques and valve lesions, thereby contributing to maladaptive tissue repair [22, 49].

## Reducing Lp(a) Level: Clinical Implications

Evidence supporting therapies that specifically target Lp(a) is rapidly emerging. Given that Lp(a) is estimated to be five- to six-fold more atherogenic than LDL cholesterol, lowering its concentration is expected to improve prognosis in patients with ASCVD and elevated Lp(a) [42, 43]. Whether this translates into a reduction in cardiovascular events, however, awaits confirmation from ongoing outcome trials. Current guidelines recommend at least one lifetime measurement of Lp(a) in all adults, with earlier testing in children or adolescents who have risk factors such as familial hypercholesterolemia, a family history of premature ASCVD, or markedly elevated levels. Testing is also advised in patients with recurrent cardiovascular events without an apparent cause [49–52]. Because Lp(a) levels are genetically determined and generally stable over time, a single measurement is often sufficient to identify high-risk individuals before their first ASCVD event [49–52]. This approach may provide both long-term clinical and economic benefits by enabling earlier prevention.

Even in the absence of definitive trial data, elevated Lp(a) remains clinically actionable. Patients with high concentrations may benefit from more intensive lifestyle interventions, optimized lipid-lowering therapy, and, in selected cases, lipoprotein apheresis [52]. Statins remain the cornerstone of ASCVD prevention despite paradoxically increasing Lp(a) levels, likely through upregulation of *LPA* expression and apo(a) synthesis [53], because their overall cardiovascular benefit outweighs this effect. PCSK9 inhibitors have demonstrated modest Lp(a)-lowering effects, particularly in patients with higher baseline levels, but their role is limited by a lack of regulatory approval for this indication [54, 55]. Aspirin has shown preventive benefit in individuals with elevated Lp(a), even though such benefit is absent in the general population [56, 57]. Lipoprotein apheresis remains a valuable option for select high-risk patients, acutely lowering both LDL cholesterol and Lp(a); however, its cost, invasiveness, and transient effects underscore the need for scalable pharmacological alternatives [58–60].

### Incorporating Lp(a) Testing and Therapy into Clinical Practice

From a practical standpoint, Lp(a) measurement can be embedded into routine cardiovascular risk evaluation, with once-in-lifetime testing recommended for all adults and targeted earlier testing in those with premature ASCVD, familial hypercholesterolemia, or unexplained recurrent events. Levels ≥ 50 mg/dL (≥ 125 nmol/L) are generally considered elevated and should prompt intensified control of all modifiable risk factors, including LDL-C lowering to guideline-recommended thresholds. For patients with very high levels (> 180 mg/dL) or progressive ASCVD, referral to specialized centers for consideration of lipoprotein apheresis or enrollment in trials of novel RNA-based therapies may be appropriate.

### Differences in Primary vs. Secondary Prevention

The clinical role of Lp(a) testing differs by prevention stage. In primary prevention, Lp(a) serves primarily as a risk-enhancer, reclassifying patients at borderline or intermediate risk and justifying earlier or more intensive statin therapy. In secondary prevention, elevated Lp(a) identifies residual genetic risk in patients with established ASCVD and supports the need for aggressive LDL-C lowering, including PCSK9 inhibitor therapy and potential apheresis in refractory cases. Thus, while testing in primary prevention refines risk estimation, testing in secondary prevention informs treatment intensification and specialized referral.

### Aspirin in Primary Prevention with Elevated Lp(a)

A particular area of controversy is aspirin use in patients with elevated Lp(a). Subgroup and observational studies suggest disproportionate benefit in this population, likely because of Lp(a)’s prothrombotic properties. Yet, aspirin is no longer routinely recommended for primary prevention given the increased risk of bleeding and the absence of dedicated randomized trials in high-Lp(a) patients [56, 57]. In clinical practice, aspirin may be considered selectively in individuals with markedly elevated Lp(a), borderline to intermediate ASCVD risk, and low bleeding risk, following shared decision-making. This individualized approach balances the potential vascular benefits against bleeding hazards until more definitive evidence emerges.

The extent of Lp(a) reduction required to meaningfully lower event rates remains uncertain. Mendelian randomization studies suggest that large absolute reductions, approximately 105–210 nmol/L (50–100 mg/dL), may be necessary [61–63]. Novel investigational therapies—including RNA-targeted agents such as pelacarsen, olpasiran, zerlasiran, and lepodisiran, as well as small-molecule inhibitors like muvalaplin—have demonstrated > 80–90% reductions in Lp(a) in phase 2 studies and also lower oxidized phospholipids and systemic inflammation, amplifying their potential benefit [61–63]. Importantly, observational studies have hinted at a possible association between very low Lp(a) levels and an increased risk of type 2 diabetes, highlighting the need for careful long-term safety assessment [64].

Overall, the integration of novel Lp(a)-lowering therapies into clinical practice will depend not only on the demonstration of outcome benefit in ongoing phase 3 trials but also on their safe, cost-effective, and targeted use. Personalized approaches that consider baseline Lp(a) level, comorbidities, and residual cardiovascular risk will be essential for optimizing the clinical and socioeconomic impact of these promising therapies.

## Clinical Trials and Emerging Management Strategies

Recommendations for Lp(a) testing and risk assessment have only recently been incorporated into clinical practice, largely in response to accumulating evidence of its pathogenic role. In 2010, the European Atherosclerosis Society first proposed a threshold of 50 mg/dL as a risk enhancer for estimating 10-year ASCVD risk, a cutoff later reaffirmed by multiple European and U.S. societies [23]. The 2018 American College of Cardiology and American Heart Association cholesterol guidelines recommended Lp(a) measurement in individuals with a family history of premature ASCVD or in patients with established ASCVD not fully explained by conventional risk factors [65].

In 2019, the National Lipid Association broadened these recommendations, advising testing in patients at very high ASCVD risk; those with severe hypercholesterolemia or suspected familial hypercholesterolemia; a family history of elevated Lp(a); inadequate LDL-C lowering despite therapy; recurrent ASCVD events; or calcific aortic stenosis. The National Lipid Association also endorsed testing in patients with borderline or intermediate ASCVD risk when uncertainty exists about initiating statin therapy [66]. A more recent update supported universal screening and advised repeat testing in patients with intermediate levels (75–125 nmol/L or 30–50 mg/dL), recognizing that Lp(a) may fluctuate under certain conditions such as menopause, chronic kidney disease, proteinuria, or hypothyroidism, potentially altering risk stratification [50–52].

The 2019 European Society of Cardiology dyslipidemia guidelines similarly recommended at least one lifetime Lp(a) measurement, particularly to identify individuals with very high concentrations (> 180 mg/dL), which confer risk comparable to heterozygous familial hypercholesterolemia [50–52]. For such patients, PCSK9 inhibitor therapy was suggested. The 2021 Canadian Cardiovascular Society guidelines likewise endorsed a one-time measurement as part of initial lipid screening and recommended intensifying lipid-lowering therapy, including PCSK9 inhibitors, for secondary prevention in patients with elevated Lp(a) [50]. In 2022, the European Atherosclerosis Society expanded its position, recommending Lp(a) testing in all adults and extending screening to younger individuals with ischemic stroke, premature ASCVD, or a family history of markedly elevated Lp(a) in the absence of other risk factors. Both U.S. and European societies have also suggested considering lipoprotein apheresis in patients with very high Lp(a) and progressive ASCVD despite optimal risk factor management [51, 67].

Alongside evolving guidelines, multiple therapeutic strategies have demonstrated the ability to lower plasma Lp(a). Distinct drug classes with diverse mechanisms of action and pharmacokinetic properties are under investigation, with Lp(a)-targeted therapies emerging as a major frontier in cardiovascular prevention. Several agents are already being evaluated in phase 1–3 clinical trials (Table 1), while additional novel compounds are progressing through earlier stages of development and are expected to enter clinical testing in the near future.

**Table 1:** Lp(a)-targeting drugs in advanced clinical trials

Drug/Type	Mechanism of Action	Elimination half-life	T_max_	Route of Administration	Elimination	Lp(a) changes in treatment arm(s)
Pelacarsen/ASO	mRNA degradation	3–4 weeks	2.5 h	Subcutaneous	Renal	–35% (20 mg/4 weeks) –56% (40 mg/4 weeks) –58% (20 mg/2 weeks) –72% (60 mg/4 weeks) –80% (20 mg/week)
Olpasiran/siRNA	mRNA degradation	~ 5.5 h	~ 7.5 h	Subcutaneous	Renal	–66.9% (10 mg) –93.8% (75 mg) –97.5% (225 mg/12 weeks) –96.9% (225 mg/24 weeks)
Zerlasiran/siRNA	mRNA degradation	~ 3.5 h	~ 2 h	Subcutaneous	Renal	–30% (300 mg) –29% (600 mg) –97% (2 doses of 200 mg) –98% (2 doses of 300 mg) –99% (2 doses of 450 mg)
Lepodisiran/siRNA	mRNA degradation	70–414 h	~ 10.5 h	Subcutaneous	Renal	–41% (4 mg) –59% (12 mg) –76% (32 mg) –90% (96 mg) –96% (304 mg) –97% (608 mg)
Muvalaplin/ Small molecule	inhibits interaction between apo(a) & apo B	12.1–414 h	2–6 h	Oral	Not reported	–48% (10 mg/day) –82% (60 mg/day) –86% (240 mg/day)
Obicetrapib/ Small molecule	CETP inhibition	81–166 h	4 h	Oral	Fecal	–32.5% (5 mg) –56.5% (10 mg)

### Pelacarsen (TQJ230, ISIS 681257, IONIS-APO[a]-LRx, AKCEA-APO[a]-LRx)

Antisense oligonucleotides (ASOs) are short, single-stranded synthetic RNA or DNA molecules that bind complementary nucleotide sequences to modulate gene expression. The first ASO specifically developed to target Lp(a) mRNA and lower Lp(a) levels was IONIS-APO(a)Rx, a second-generation compound incorporating five 2ʹ-MOE–modified ribonucleosides at each end and ten 2ʹ-deoxyribonucleosides in the central region [68]. In a Phase 1 trial of 47 healthy adults with Lp(a) ≥ 25 nmol/L (≥ 10 mg/dL), a single subcutaneous dose of IONIS-APO(a)Rx (50–400 mg) produced no significant effect on Lp(a) at Day 30. However, multiple dosing (six injections of 100–300 mg on Days 1, 3, 5, 8, 15, and 22) achieved reductions of 40–78% [66]. A subsequent Phase 2 study (*N* = 64) enrolled participants with baseline Lp(a) of 125–437 nmol/L (Cohort A) or ≥ 438 nmol/L (Cohort B). Weekly administration of escalating doses (100, 200, or 300 mg) for four weeks lowered Lp(a) by 67% in Cohort A and 72% in Cohort B (both *P* < 0.0001) [69].

Pelacarsen is a GalNAc3-conjugated derivative of IONIS-APO(a)Rx designed to enhance hepatocyte-specific uptake via the asialoglycoprotein receptor following subcutaneous administration [70]. In preclinical mouse studies, GalNAc3 conjugation increased potency approximately 20-fold for hepatic apo(a) mRNA knockdown and plasma apo(a) protein reduction compared with the unconjugated ASO. Pelacarsen also demonstrated rapid plasma clearance, tissue conversion to the unconjugated ASO, and a half-life of 7–8 days [71].

To date, pelacarsen has been tested in three randomized, placebo-controlled, double-blind trials. In a Phase 1/2a study, 58 healthy participants with Lp(a) ≥ 75 nmol/L received either a single dose (10–120 mg) or multiple ascending doses (10, 20, or 40 mg) over 22 days. In the single-dose cohorts, mean Lp(a) reductions at Day 30 ranged from 25% (10 mg) to 85% (120 mg). In the multiple-dose cohorts, reductions at Day 36 ranged from 59% (10 mg) to 82% (40 mg). Sustained reductions versus placebo persisted through Day 90 in the 80 mg (46%) and 120 mg (44%) single-dose groups (both *P* < 0.05) and through Day 113 across all multiple-dose groups (39%, 53%, and 58% reductions with 10, 20, and 40 mg, respectively). Compared with the non-GalNAc3–conjugated IONIS-APO(a)Rx, pelacarsen exhibited a ~ 30-fold increase in potency, with a median effective dose of 3.96 mg/week versus 122 mg/week [72].

A Phase 2b trial investigated pelacarsen in 286 patients with established CVD and baseline Lp(a) ≥ 60 mg/dL (≥ 150 nmol/L). Participants received pelacarsen at 20, 40, or 60 mg every 4 weeks (Q4W), 20 mg every 2 weeks (Q2W), or 20 mg weekly (QW) versus placebo for 6–12 months. At 6 months, pelacarsen significantly reduced Lp(a) levels by 35% (20 mg Q4W) to 80% (20 mg QW), compared with a 6% reduction in the placebo group (all *P* < 0.005). Notably, equivalent reductions were observed across regimens delivering the same cumulative dose but at different dosing intervals—for example, 56% with 40 mg Q4W versus 58% with 20 mg Q2W [73]. Reductions were evident within the first month, reached near-maximal effect by Week 16, and returned to baseline within 16 weeks of treatment discontinuation. After 6 months, the proportion of patients achieving Lp(a) ≤ 50 mg/dL ranged from 23% (20 mg Q4W) to 98% (20 mg QW), while those achieving ≤ 30 mg/dL ranged from 6% to 71%, respectively [73].

In a separate Phase 1 trial in Japanese individuals (*N* = 29), participants received a single dose of 20, 40, or 80 mg pelacarsen, or placebo, or multiple doses of 80 mg Q4W on Days 1, 29, 57, and 85. By Day 30, dose-dependent, placebo-corrected least-square mean (LSM) reductions in Lp(a) were observed across all single-dose groups (55–74%, all *P* < 0.001). In the 80 mg Q4W multiple-dose cohort, placebo-corrected LSM reductions were 84%, 106%, 70%, 80%, and 56% on Days 29, 85, 113, 176, and 204, respectively. Pharmacokinetic analyses showed that plasma pelacarsen peaked at ~ 4 h post-dose, with a mean elimination half-life of ~ 5 weeks following multiple 80 mg Q4W administrations [74].

In the Phase 1/2a trial, pelacarsen demonstrated a favorable safety profile, with no injection-site reactions, serious adverse events (SAEs), influenza-like symptoms, or other safety concerns, and no study discontinuations [68–74]. In the Phase 2b trial, adverse events occurred in 90% of patients receiving pelacarsen and 83% of those on placebo, the majority being mild or moderate in severity. SAEs were reported in 10% of pelacarsen-treated patients versus 2% in the placebo group, without evidence of a dose-dependent relationship. Discontinuation rates were comparable between groups (5% vs. 4%, respectively) [68–74]. Similarly, in the Phase 1 trial of healthy Japanese participants, no treatment-related AEs leading to discontinuation, no predefined AEs of special interest, and no deaths were observed. Long-term safety data are awaited from the ongoing Phase 3 program.

The pivotal Phase 3 Lp(a)HORIZON trial (NCT04023552) has completed enrollment (*N* = 8,324; October 2022) [75]. This trial is evaluating pelacarsen 80 mg Q4W (SC) in patients with established CVD and baseline Lp(a) ≥ 70 mg/dL, with the primary objective of determining whether targeted Lp(a) reduction lowers the incidence of major adverse cardiovascular events (MACE). Completion is anticipated in 2026, marking the first Phase 3 study of an RNA-based Lp(a)-lowering therapy designed to assess clinical outcome benefit. Additional trials are ongoing, including the Phase 2 Lp(a)FRONTIERS CAVS study (NCT05646381), which is examining the effect of pelacarsen on progression of calcific aortic valve stenosis, and a Phase 3b trial evaluating Lp(a)-lowering efficacy and safety in Black and Hispanic patients in the U.S. with ASCVD and elevated Lp(a) (≥ 125 nmol/L).

### Olpasiran (AMG890), Zerlasiran (SLN360), and Lepodisiran (LY3819469)

The discovery of endogenous RNA interference (RNAi) revealed key mechanisms regulating protein expression and opened the door to developing therapeutics capable of mimicking this process in a targeted manner. The advent of siRNA agents has leveraged this principle, using GalNAc conjugation to selectively deliver these molecules to the liver, where they can modulate atherogenic lipid factors. Currently, three siRNAs targeting Lp(a) are under clinical investigation: olpasiran (AMG890), zerlasiran (SLN360), and lepodisiran (LY3819469) (Table 2) [76–78]. Administered subcutaneously, these agents reduce Lp(a) levels in a dose-dependent manner, achieving approximately 40% reduction at lower doses and up to 99% at higher doses (e.g., 225 mg for olpasiran, 600 mg for zerlasiran, and 608 mg for lepodisiran). Their prolonged pharmacodynamic effects allow for infrequent dosing; for example, olpasiran can be administered once every 3–6 months. Overall, these therapies have shown favorable tolerability, with predominantly mild adverse events, including injection-site reactions, hypersensitivity, headache, and upper respiratory tract infections.

**Table 2:** Comparison among Olpasiran (AMG 890), Zerlasiran (SLN360), and lepodisiran (LY3819469)

Feature	Olpasiran (AMG 890)	Zerlasiran (SLN360)	Lepodisiran (LY3819469)
Modality/target	GalNAc-conjugated short interfering RNA (siRNA) targeting *LPA* mRNA.	GalNAc-conjugated siRNA targeting *LPA* mRNA.	GalNAc-conjugated siRNA targeting *LPA* mRNA.
Structure/chemistry	Double-stranded ~ 21-nt siRNA backbone with chemical stabilizations (2’ modifications, PS linkages) and a triantennary GalNAc ligand for hepatocyte uptake.	GalNAc-siRNA design: short dsRNA with chemical modifications for nuclease resistance and a GalNAc targeting ligand.	Chemically stabilized GalNAc-conjugated siRNA (2’ modifications and GalNAc targeting).
Plasma T_max_	~ 7.5 h	~ 2 h	~ 10.5 h
Plasma half-life	~ 5.5	~ 3.5	70–414 h
Pharmacodynamics — Lp(a) lowering & duration	Potent Lp(a) lowering (Phase-2 OCEAN(a) DOSE: ≥~70% up to > 95% reductions at week 36 for higher doses; higher doses produced > 95% reductions at 36 wks with durable off-treatment effects). Durable effect — partial Lp(a) reductions persist months after dosing.	Very large reductions in Lp(a) in APOLLO/ALPACAR: median reductions up to ~ 96–98% at high doses; reductions of ~ 80–85% persisted. over many weeks.	Very large and durable reductions: Phase-1/2 data show ~ 94% reductions (e.g., 93.9% averaged day 60–180 at 400 mg in ALPACA/phase-2), with effects lasting many months.
Dosing regimen used in trials	SC injections (examples: 10, 75, 225 mg q12w or q24w in OCEAN(a)-DOSE; higher doses q12w produced maximal reductions).	Single-dose cohorts (30–600 mg) in phase-1; phase-2 schedules studied multiple dosing intervals (e.g., 300 mg q16w or q24w, or 450 mg q24w in ALPACAR).	Single-dose cohorts in phase-1 (4–608 mg); phase-2 used regimens such as 400 mg (single or two doses with spacing); durable effect supports infrequent dosing.
Development/clinical status	Advanced: Phase-2 (OCEAN(a) DOSE completed; overall positive Lp(a) lowering; cardiovascular outcomes trials planned/ongoing).	Advanced: Phase-1/2 completed; Phase-3 planning/ongoing.	Advanced: Phase-1 & phase-2 positive results (ALPACA), large phase-3/outcomes studies in planning/ongoing.

Olpasiran was evaluated in a dose-finding trial involving patients with established ASCVD and baseline Lp(a) > 150 nmol/L. After 36 weeks of treatment, placebo-adjusted Lp(a) reductions were 70.5% with 10 mg every 12 weeks, 97.4% with 75 mg every 12 weeks, 101.1% with 225 mg every 12 weeks, and 100.5% with 225 mg every 24 weeks. The therapy was generally well tolerated, with injection-site reactions being the most common adverse event [76]. Long-term follow-up demonstrated that Lp(a) lowering exceeding 40% persisted for more than 12 months after the final 225 mg dose [63]. The OCEAN(a)-Outcomes trial is currently investigating the effect of olpasiran versus placebo administered every 12 weeks on the composite incidence of coronary death, myocardial infarction, or urgent coronary revascularization. The study is enrolling 7,000 patients aged 18–85 years with a history of ASCVD and Lp(a) ≥ 200 nmol/L [79].

Zerlasiran is a 19-mer siRNA conjugated to a tri-antennary GalNAc moiety for targeted hepatic delivery. In an early single-ascending dose study in adults with Lp(a) ≥ 150 nmol/L, zerlasiran was well tolerated and produced dose-dependent reductions in Lp(a) of 46% (30 mg), 86% (100 mg), 96% (300 mg), and 98% (600 mg), with effects persisting for up to 150 days, compared with a 10% reduction in the placebo group [80]. In a multiple-dose study of patients with established ASCVD and Lp(a) ≥ 150 nmol/L, maximal Lp(a) reductions were observed at 60% (200 mg), 90% (300 mg), and 89% (450 mg). The effect was durable, with persistent reductions of 60%, 90%, and 89% maintained for 201 days post-administration of each respective dose [81].

Lepodisiran is a Dicer-substrate siRNA with a tetraloop structure incorporating three GalNAc conjugates for targeted liver delivery. In an early single-ascending dose study in adults without cardiovascular disease and with Lp(a) ≥ 75 nmol/L, lepodisiran was well tolerated and produced dose-dependent reductions in Lp(a) of 41% (4 mg), 59% (12 mg), 76% (32 mg), 90% (96 mg), 96% (304 mg), and 97% (608 mg) at Day 148, compared with a 5% reduction with placebo. Remarkably, Lp(a) levels remained 94% below baseline 337 days after the 608 mg dose [82]. Lepodisiran is now being evaluated in larger, longer-term studies and has advanced to a cardiovascular outcomes trial enrolling 12,500 patients with Lp(a) ≥ 175 nmol/L. Participants include individuals with established ASCVD (age > 18 years) or high-risk primary prevention populations, defined as (1) subclinical atherosclerotic disease, (2) familial hypercholesterolemia, or (3) multiple risk factors in adults > 55 years. The trial will assess the effect of lepodisiran on the composite endpoint of cardiovascular death, myocardial infarction, stroke, or coronary revascularization. Compared with antisense oligonucleotides, siRNA agents like lepodisiran achieve more substantial and durable Lp(a) lowering, allowing less frequent dosing. Whether near-complete reduction of Lp(a) translates into greater clinical benefit without safety concerns remains to be established in ongoing outcome trials [83].

### Muvalaplin (LY3473329)

**Fig. 3:**
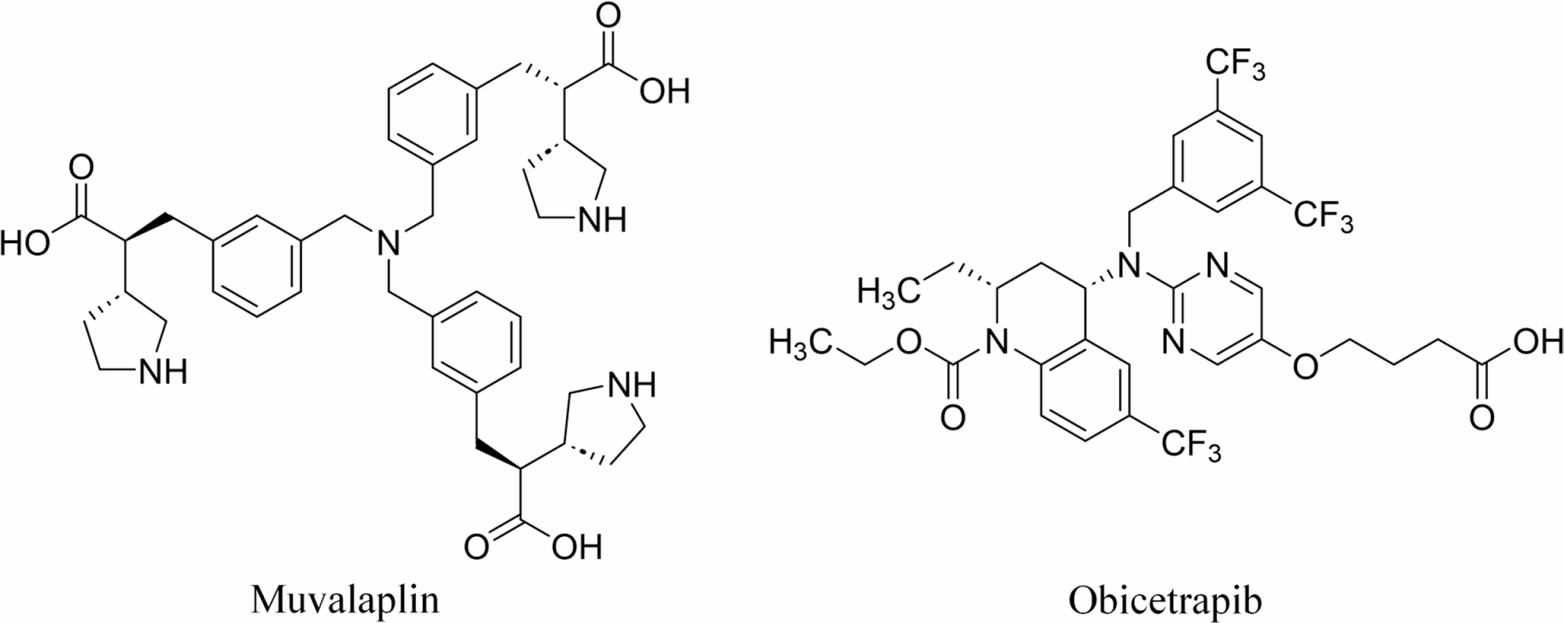
Chemical structures of small molecules that reduce Lp(**a**) levels

Muvalaplin (Fig. 3) is the first oral small-molecule inhibitor designed to lower lipoprotein(a) levels. Lp(a) biosynthesis occurs in two steps: initially, apolipoprotein(a) kringle IV domains 7 and 8 bind noncovalently to lysine residues on apolipoprotein B, followed by the formation of a covalent disulfide bond. Muvalaplin selectively targets the kringle IV domains 7 and 8 of apolipoprotein(a), disrupting the initial noncovalent interaction with apolipoprotein B and thereby preventing Lp(a) particle assembly. This mechanism mirrors naturally occurring apo(a) variants that fail to bind apolipoprotein B, which are associated with low Lp(a) levels. Preclinical studies demonstrated that muvalaplin inhibits Lp(a) assembly in vitro and effectively reduces Lp(a) concentrations in nonhuman primates [84].

In the first-in-human, placebo-controlled Phase 1 study, muvalaplin was administered for up to 14 days and demonstrated good tolerability with no serious adverse events. Daily dosing produced dose-dependent reductions in Lp(a), reaching up to 65% by Day 14. Lp(a) levels remained lowered for up to 50 days after the final dose. Among participants with elevated baseline Lp(a), the highest dose resulted in 93% achieving trough Lp(a) levels ≤ 50 mg/dL by the end of treatment. Muvalaplin had no significant effects on other lipid parameters in healthy participants with elevated Lp(a) [85].

In a Phase 2 study, muvalaplin continued to show good tolerability and effective Lp(a) lowering. At the highest dose tested (240 mg), muvalaplin achieved a placebo-adjusted reduction of intact Lp(a) by 85.8% and a reduction of Lp(a) measured by a traditional apo(a)-based assay by 68.9%. At this dose, 96.7% of patients reached Lp(a) levels < 125 nmol/L using the intact Lp(a) assay and 77.4% using the apo(a) assay. Dose-dependent reductions in LDL-C (up to 21.3%) and apoB (up to 16.1%) were also observed. Overall, muvalaplin was well tolerated, with no dose-dependent adverse events or concerning biochemical abnormalities [86].

### Obicetrapib (TA-8995)

CETP is a glycoprotein synthesized in the liver that mediates lipid exchange between lipoproteins. It facilitates the transfer of cholesteryl esters from HDL to LDL and VLDL, while simultaneously moving triglycerides from VLDL and chylomicrons to HDL and LDL. Two mechanisms have been proposed to explain this process: the shuttle mechanism, where CETP binds to a single lipoprotein particle, completes lipid transfer, and detaches before repeating the process; and the tunnel mechanism, where CETP bridges HDL with LDL or VLDL to create a channel that enables bidirectional lipid movement. Both pathways ultimately redistribute lipids in ways that deplete HDL and enrich LDL and VLDL, patterns linked with atherogenesis [87].

Clinical development of CETP inhibitors has been marked by mixed outcomes. Torcetrapib, the first large-scale candidate, raised HDL-C by approximately 70% and lowered LDL-C by about 25% [88]. However, it was associated with increased mortality and adverse cardiovascular events, attributed to off-target effects such as elevated blood pressure, electrolyte disturbances, and stimulation of aldosterone and cortisol. Dalcetrapib produced more modest effects, raising HDL-C by around 30% with negligible impact on LDL-C. Despite being well tolerated, it failed to show any reduction in major cardiovascular events [89]. Evacetrapib combined strong lipid effects with safety but did not reduce cardiovascular outcomes during a median follow-up of just over two years, a duration thought to be too short to capture long-term benefits [87]. Anacetrapib provided the first convincing evidence that CETP inhibition could reduce cardiovascular risk. In a large trial involving over 30,000 patients followed for more than four years, it reduced major coronary events by nearly 10% during the study, and benefits roughly doubled in the years following completion. The main drawback was drug accumulation in adipose tissue, which raised concerns about long-term use despite an otherwise favorable safety profile [90]. The most recently developed CETP inhibitor is obicetrapib.

Obicetrapib is a tetrahydroquinoline derivative (Fig. 3) containing pyrimidine and ethoxycarbonyl groups and featuring two chiral centers. Compared with CETP inhibitors such as anacetrapib and evacetrapib, obicetrapib demonstrates more potent CETP inhibition, likely due to its lower lipophilicity and greater polarity, which may enhance binding affinity, specificity, and solubility. Crystallographic studies indicate that CETP inhibitors generally bind the N-terminal hydrophobic tunnel of CETP to block lipid transfer, and obicetrapib’s increased polarity may facilitate additional interactions with central polar residues [91]. Preclinical studies supported its progression to clinical development, which includes multiple Phase 1 trials in healthy volunteers, at least six completed Phase 2 trials in patients with dyslipidemia or elevated Lp(a), and three ongoing Phase 3 trials. Furthermore, obicetrapib is being investigated for Alzheimer’s disease in a Phase 2a proof-of-concept study.

Phase 1 studies of obicetrapib showed that the drug was well tolerated, with no clinically meaningful effects on vital signs, blood pressure, or laboratory parameters, including aldosterone, sodium, potassium, and bicarbonate, which had been concerns with earlier CETP inhibitors [92]. Daily doses ranging from 2.5 to 25 mg achieved near-complete CETP inhibition (92–99%), increased HDL-C by 96–140%, and reduced LDL-C by 40–53%. Pharmacokinetic and pharmacodynamic profiles were consistent across age, sex, ethnicity, and fed versus fasted conditions, supporting broad applicability. Additional Phase 1 investigations provided further insights into safety and pharmacology. A thorough QT study confirmed no effect on the heart rate–corrected QT interval. Drug-drug interaction studies indicated that obicetrapib is a mild CYP3A4 inducer without significant impact on P-glycoprotein activity. Mass balance and metabolism studies demonstrated steady absorption, with fecal excretion as the primary elimination route. Switching from capsule to tablet formulations confirmed bioequivalence at the 5 mg dose, and food studies showed 55–59% higher exposure under fed conditions, suggesting administration with or without food is feasible. Pharmacokinetic assessments in Chinese subjects mirrored those in Caucasian volunteers, confirming consistency across populations. A pilot study of fixed-dose combinations with ezetimibe demonstrated bioequivalence for total exposure in one formulation, although maximum concentration criteria were not fully met. Additional Phase 1 trials are planned to further evaluate obicetrapib’s pharmacodynamics, including its effect on the fractional catabolic rate of ApoB in LDL when administered alongside statin therapy in individuals with normal or dyslipidemic lipid profiles [91].

The first Phase 2 trial of obicetrapib, TA-8995: Its Use in Patients with Mild Dyslipidaemia (TULIP), conducted in Denmark and the Netherlands, evaluated daily obicetrapib monotherapy doses of 1, 2.5, 5, or 10 mg, as well as obicetrapib 10 mg in combination with 20 mg atorvastatin or 10 mg rosuvastatin, compared with placebo or statin alone, over 12 weeks in patients with mild dyslipidemia [93, 94]. Monotherapy with 5 mg and 10 mg obicetrapib produced a significant median LDL-C reduction of 45% from baseline. At the 10 mg dose, ApoB decreased by 34%, Lp(a) by 33%, and HDL-C increased by 179%. Following these findings, a dedicated study assessed the effects of obicetrapib 2.5 mg and 10 mg administered for 12 weeks versus placebo in participants with elevated Lp(a). Both doses significantly lowered Lp(a) compared with placebo, although the reductions were smaller than those observed in previous studies. Another dose-finding study in Japanese participants evaluated obicetrapib 2.5, 5, and 10 mg over 8 weeks as an adjunct to stable statin therapy (atorvastatin 10 or 20 mg, or rosuvastatin 5 or 10 mg). At the 10 mg dose, median LDL-C decreased by 46%, ApoB by 30%, and HDL-C increased by 159% [91].

The combination of obicetrapib with high-intensity statins and ezetimibe has been assessed in three Phase 2 trials in patients with dyslipidemia: obicetrapib added to high-intensity statins (ROSE) [95], obicetrapib combined with ezetimibe (OCEAN), and obicetrapib plus ezetimibe on top of high-intensity statins (ROSE2) [96]. In ROSE, administration of 5 mg and 10 mg obicetrapib for 8 weeks alongside high-intensity statins led to significant median reductions in LDL-C (up to 51%), ApoB (up to 30%), and non-HDL-C (up to 44%), while HDL-C increased by up to 165% [95]. OCEAN showed that 5 mg obicetrapib alone reduced LDL-C by 34%, and when combined with 10 mg ezetimibe, reductions reached 52% after 8 weeks. In ROSE2, patients received 10 mg obicetrapib alone or in combination with 10 mg ezetimibe for 12 weeks on top of high-intensity statins. This regimen produced significant reductions in LDL-C, non-HDL-C, ApoB, total and small LDL particles, and Lp(a), while HDL-C increased by up to 142%. Among patients receiving the combination of obicetrapib and ezetimibe with high-intensity statins, 100% achieved LDL-C < 100 mg/dL, 93.5% achieved < 70 mg/dL, and 87.1% achieved < 55 mg/dL [96]. These findings highlight obicetrapib’s potential to address the treatment gap for patients with elevated LDL-C who fail to reach target levels with currently available therapies [97–99].

Obicetrapib has been investigated in multiple Phase 3 trials. BROADWAY enrolled over 2,500 patients with established ASCVD who require additional LDL-C lowering. BROOKLYN has completed enrollment of 354 participants with heterozygous familial hypercholesterolemia across ten countries. PREVAIL aims to recruit 9,000 participants to evaluate whether obicetrapib reduces major adverse cardiovascular events, including cardiovascular death, non-fatal myocardial infarction, non-fatal stroke, and non-elective coronary revascularization, in patients with ASCVD whose LDL-C remains inadequately controlled on maximal statin therapy. The BROADWAY trial was a multinational, randomized, placebo-controlled study assessing 10 mg daily obicetrapib in 2,530 high-risk patients, including those with heterozygous familial hypercholesterolemia or established ASCVD, all receiving maximally tolerated lipid-lowering therapy. The primary endpoint was the percent change in LDL-C from baseline to Day 84. Obicetrapib reduced LDL-C by 29.9% compared with a 2.7% increase in the placebo group, corresponding to a between-group difference of − 32.6% points (*P* < 0.001). Adverse event rates were comparable between groups, demonstrating a favorable safety profile and confirming obicetrapib’s effectiveness in lowering LDL-C in high-risk patients already on standard therapy [100].

The TANDEM trial was a Phase 3, randomized, double-blind, placebo-controlled study evaluating a fixed-dose combination of obicetrapib 10 mg and ezetimibe 10 mg in 407 adults at high risk for ASCVD or with heterozygous familial hypercholesterolemia. Participants received the combination, obicetrapib alone, ezetimibe alone, or placebo daily for 84 days. At Day 84, the fixed-dose combination reduced LDL-C by 48.6% versus placebo, 27.9% versus ezetimibe alone, and 16.8% versus obicetrapib monotherapy, while obicetrapib alone lowered LDL-C by 31.9% versus placebo. Adverse events were similar across groups, with serious events and deaths infrequent and balanced. The study concluded that the obicetrapib-ezetimibe fixed-dose combination is highly effective, well-tolerated, and offers a convenient oral option to improve LDL-C management in high-risk patients [101].

### CTX320

CRISPR technology represents a fundamentally different approach from antisense oligonucleotides and siRNA. Whereas those agents require repeated dosing to silence *LPA* mRNA, CRISPR-based therapies aim for permanent inactivation of the *LPA* gene in hepatocytes through targeted DNA editing. CTX320 is the most advanced investigational candidate in this space. It is a CRISPR/Cas9-based gene-editing therapy specifically designed to disable the apo(a) component responsible for Lp(a) production in the liver. The therapy uses lipid nanoparticles to deliver Cas9 mRNA and a guide RNA directly to hepatocytes, with the intent of achieving a single, durable reduction in Lp(a). In preclinical studies with non-human primates, CTX320 demonstrated dose-dependent reductions in plasma Lp(a) of approximately 20%, 80%, and 90% at doses of 0.5, 1.5, and 3 mg/kg, respectively. In another ongoing study, a single 2 mg/kg infusion achieved a ~ 94% reduction in Lp(a) within 14 days, and this effect was sustained through Day 224. Importantly, treatment was well tolerated, with no major safety signals, supporting its advancement into phase I clinical trials [102–104].

Potential advantages of CTX320 and similar CRISPR-based therapies include the possibility of a one-time curative intervention, elimination of adherence challenges, and profound, durable Lp(a) suppression. However, significant challenges remain, including risks of off-target editing, long-term safety, immune responses to delivery vectors, manufacturing complexity, and extremely high costs. At present, clinical experience in humans is lacking, and it remains to be determined whether this approach will prove both safe and clinically effective.

## Conclusion Remarks

Lp(a) is a genetically determined, independent contributor to ASCVD [4, 5, 11, 105, 106]. Its structural complexity and strong genetic regulation complicate accurate measurement and standardized reporting. A range of novel therapies aimed at lowering Lp(a) is in development, including siRNAs, ASOs, small molecules, and CRISPR/Cas9-based approaches, each characterized by unique mechanisms, pharmacokinetics, and stages of clinical testing. Research is actively exploring how these interventions reduce Lp(a) and their potential to mitigate ASCVD risk [107, 108].

Concurrently, clinical practice regarding Lp(a) testing continues to evolve, with guidelines and consensus statements increasingly emphasizing its role in cardiovascular risk assessment. Ongoing Phase 3 trials are assessing whether Lp(a) reduction leads to significant long-term cardiovascular benefits, with results from pelacarsen anticipated early next year and olpasiran expected by the end of 2026 (Table 3). These studies will provide critical insight into the therapeutic value of Lp(a) lowering across diverse patient populations.

**Table 3:** Ongoing phase 3 double blind trials of drugs targeting Lp(a)

Trial	ACCLAIM-Lp(a) (NCT06292013)	Lp(a)HORIZON (NCT04023552)	OCEAN(a)–Outcomes Trial (NCT05581303)
Investigational drug	Lepodisiran (SubQ)	Pelacarsen (SubQ/month)	Olpasiran (SubQ/12 weeks)
Population	Individuals with Lp(a) > 175 nmol/L and at high risk of cardiovascular events or with established ASCVD	Individuals with established ASCVD and Lp(a) > 175 nmol/L	Individuals with Lp(a) > 200 nmol/L, a history of ASCVD (previous type 1 MI or previous revascularization with PCI), and at least 1 prespecified risk-enhancing feature
Sample	*N* = 12 500	*N* = 8323	*N* = 7297
Primary outcome(s)	Time to first major adverse cardiac event (MACE, cardiovascular death, MI, stroke, or urgent coronary revascularization)	Time to first MACE	Time to first MACE

A single measurement of Lp(a) for all individuals could help identify those at increased cardiovascular risk and prevent a significant number of events, with the anticipated benefits likely exceeding the costs of screening and intervention. In the absence of definitive trial data, clinicians operate in a “gray zone” and are encouraged to manage modifiable risk factors aggressively in patients with elevated Lp(a). This approach includes optimizing LDL-C lowering, considering aspirin therapy when appropriate, and controlling hypertension, obesity, and diabetes. Testing first-degree relatives is advised, and motivated patients may be invited to participate in clinical trials. Awareness of Lp(a) among healthcare providers is steadily increasing, leading to wider testing, though systemic challenges, such as limited insurance coverage, gaps in physician education, and logistical hurdles, persist. Despite these barriers, progress is moving toward a future where Lp(a) measurement and targeted interventions are a routine part of ASCVD prevention.

Lastly, the available data must be interpreted with caution. Several studies are small, single-center trials, which restrict the general application of the findings. Furthermore, many studies rely on surrogate endpoints, making it difficult to draw definitive conclusions about long-term benefits. Safety concerns, including adverse event profiles and potential drug interactions, are not yet fully characterized, particularly in specific patient populations. These limitations underscore the need for larger, well-designed randomized trials before widespread adoption in clinical practice.

## Data Availability

Included in the manuscript.
